# Supplementary material for: Appraisal of Intraoperative Adverse Events to Improve Postoperative Care
Source: J Clin Med. 2023 Mar 28;12(7):2546. doi: 10.3390/jcm12072546 (PMC10095268; doi:10.3390/jcm12072546)
Supplement: Supplementary file 1 [file jcm-12-02546-s001.zip › jcm-2217053-supplementary.pdf]

Supplementary Materials - Index

**Table S1**	ClassIntra version 1.0 classification of intraoperative adverse events.	p. 2
**Table S2**	Multivariable linear mixed model of CCI - comparison of models with only ClassIntra® and ClassIntra® including origin of intraoperative adverse event.	p. 3
**Table S3**	Description of other surgical and anesthesia iAEs	p. 4

Supplementary Tables

Table S1 ClassIntra version 1.0 classification of intraoperative adverse events. The classification defines intraoperative adverse events as any deviation from the ideal intraoperative course occurring between skin incision and skin closure. Any event related to surgery and anaesthesia during the index surgery must be considered and should be rated directly after surgery. A requirement is that the indication for surgery and the interventions conform to current guidelines. *

Grade	Definition	Examples
**Grade 0**	No deviation from the ideal intraoperative course	
**Grade I**	Any deviation from the ideal intraoperative course:<br>· Without the need for any additional treatment or intervention<br>· Patient with no or mild symptoms	· Bleeding: Bleeding above average from small calibre vessel: self-limiting or definitively manageable without additional treatment than routine coagulation<br>· Injury: Minimal serosal lesion of intestines, not requiring any additional treatment<br>· Cautery: Small burn of the skin, no treatment necessary<br>· Arrhythmia: Arrhythmia (e.g., extrasystoles) without relevance
**Grade II**	Any deviation from the ideal intraoperative course:<br>· With the need for any additional minor treatment or intervention<br>· Patient with moderate symptoms, not life-threatening and not leading to permanent disability	· Bleeding: Bleeding from medium calibre artery or vein, ligation; use of tranexamic acid<br>· Injury: Non-transmural intestinal lesion requiring suture(s)<br>· Cautery: Moderate burn requiring non-invasive wound care<br>· Arrhythmia: Arrhythmia requiring administration of antiarrhythmic drug, no haemodynamic effect
**Grade III**	Any deviation from the ideal intraoperative course:<br>· With the need for any additional moderate treatment or intervention<br>· Patient with severe symptoms, potentially life-threatening or potentially leading to permanent disability	· Bleeding: Bleeding from large calibre artery or vein with transient haemodynamic instability, ligation or suture; blood transfusion<br>· Injury: Transmural intestinal lesion requiring segmental resection<br>· Cautery: Severe burn requiring surgical debridement<br>· Arrhythmia: Arrhythmia requiring administration of antiarrhythmic drug, transient haemodynamic effect
**Grade IV**	Any deviation from the ideal intraoperative course:<br>· With the need for any additional major and urgent treatment or intervention<br>· Patient with life-threatening symptoms or leading to permanent disability	· Bleeding: Life-threatening bleeding with splenectomy; massive blood transfusion; ICU stay<br>· Injury: Injury of central artery or vein requiring extended intestinal resection<br>· Cautery: Life-threatening burn injury by cautery leading to fire requiring ICU treatment<br>· Arrhythmia: Arrhythmia requiring electro conversion, defibrillation or admission to the ICU
**Grade V**	Any deviation from the ideal intraoperative course with intraoperative death of the patient	

* These events were not defined as intraoperative adverse events: sequelae, failures of cure, events related to the underlying disease, incorrect site or incorrect patient surgery, or errors in indication.
Table is modified from Dell-Kuster et al., 2020.

Table S2 Multivariable linear mixed model of CCI - comparison of models with only ClassIntra® and ClassIntra® including origin of intraoperative adverse event.
* Log-likelihood ratio (LLR) test between reference model of CCI and model including origin did not show evidence for a better fit of the model including origin (p=0.15).

	**Reference model**	**Model including origin**
**Factors**	Mean difference (95% CI)	Mean difference (95% CI)
**Origin**		
Surgery		2.2 (-4.2, 8.7)
Anesthesia	-	-1.3 (-7.8, 5.3)
Organization		-4.1 (-14, 5.4)
More than one adverse event		1.7 (-5.2, 8.6)
**ClassIntra®**		
Grade I vs 0	-0.1 (-2.7, 2.6)	-0.8 (-7.2, 5.6)
Grade II vs 0	2.7 (0.6, 4.8)	1.9 (-4.4, 8.2)
Grade III vs 0	9.7 (6.6, 13)	8.7 (2.0, 16)
Grade IV vs 0	19 (11, 26)	18 (8.0, 27)
**Age** (per decade increase)	0.3 (-0.2, 0.7)	0.3 (-0.2, 0.7)
**ASA class**		
ASA II vs I	1.8 (-0.1, 3.8)	2.0 (0.0, 3.9)
ASA III vs I	6.1 (3.8, 8.4)	6.2 (3.9, 8.5)
ASA IV/V vs I	15 (11, 19)	15 (11, 19)
**Complexity of surgery**		
Minor vs Complex major	1.4 (-2.3, 5.0)	1.4 (-2.3, 5.0)
Intermediate vs Complex major	-1.4 (-3.8, 1.0)	-1.4 (-3.8, 0.9)
Major vs Complex major	-2.4 (-4.4, -0.4)	-2.3 (-4.4, -0.4)
Major + vs Complex major	0.5(-1.5, 2.5)	0.5 (-1.5, 2.5)
**Duration of surgery** (per 10 min increase)	0.4 (0.3, 0.5)	0.4 (0.3, 0.5)
**Urgency** (emergency vs planned)	5.0 (2.8, 7.2)	5.0 (2.8, 7.2)
**Wound category** (non-clean vs clean)	1.9 (0.4, 3.5)	1.9 (0.4, 3.5)
**Experience of surgical team** (per each unit decrease)	-1.0 (-2.3, 0.2)	-1.0 (-2.3, 0.4)
**Experience of anesthesia team** (per each unit decrease)	-0.6 (-1.3, 0.2)	-0.5 (-1.2, 0.2)

Table S3 Description of iAEs of the 'Subcategory Other' with origins surgery or anesthesia. This is a supplement to Table 5 of the main manuscript.

Origin	Subcategory Other
**Surgery**	Distal embolism, neurological symptoms, untraceable anatomic structure, leakage of intestinal fluids, additional colon resection, small bowel rotation, abdominal fascia closure with mesothelium, vasospasm in reconstructive surgery.
**Anesthesia**	Non systemic medical side effect, usage of wrong intravenous line, extravasation of infusion medication, vomiting, leakage of laryngeal mask, preoperative anemia requiring intraoperative transfusion, blood flow in epidural catheter, needle accident, wrongly inserted epidural catheter
